# A Novel Based-Network Strategy to Identify Phytochemicals from Radix Salviae Miltiorrhizae (Danshen) for Treating Alzheimer’s Disease

**DOI:** 10.3390/molecules27144463

**Published:** 2022-07-12

**Authors:** Bo Li, Yu-Rui Wu, Lan Li, Yu Liu, Zhu-Yun Yan

**Affiliations:** 1 State Key Laboratory of Characteristic Chinese Medicine Resources in Southwest China, School of Pharmacy, Chengdu University of Traditional Chinese Medicine, Chengdu 611137, China; libo@stu.cdutcm.edu.cn (B.L.); wuyurui@stu.cdutcm.edu.cn (Y.-R.W.); lilan@stu.cdutcm.edu.cn (L.L.); liuyu@stu.cdutcm.edu.cn (Y.L.); 2 School of Pharmacy, Sichuan College of Traditional Chinese Medicine, Mianyang 621000, China

**Keywords:** Alzheimer’s diseases, Radix Salviae Miltiorrhizae (Danshen), phytochemicals, network pharmacology, molecular docking, connectivity map, anti-apoptosis

## Abstract

Alzheimer’s disease (AD) is a common age-related neurodegenerative disease that strikes millions worldwide. Herein, we demonstrate a new approach based on network target to identify anti-AD compounds from Danshen. Network pharmacology and molecular docking were employed to establish the DS-AD network, which mainly involved apoptosis of neuron cells. Then network scoring was confirmed via Connectivity Map analysis. M308 (Danshenxinkun D) was an anti-AD candidate with a high score (*p* < 0.01). Furthermore, we conducted ex vivo experiments with H_2_O_2_-treated PC12 cells to verify the neuroprotective effect of Salvia miltiorrhiza-containing plasma (SMP), and UPLC-Q-TOF/MS and RT-qPCR were performed to demonstrate the anti-AD activity of M308 from SMP. Results revealed that SMP could enhance cell viability and level of acetylcholine. AO/EB staining and Mitochondrial membrane potential (MMP) analysis showed that SMP significantly suppressed apoptosis, which may be due to anti-oxidative stress activity. Moreover, the effects of M308 and SMP on expressions of PSEN1, DRD2, and APP mRNA were consistent, and M308 can significantly reverse the expression of PSEN1 and DRD2 mRNA in H_2_O_2_-treated PC12 cells. The strategy based on the network could be employed to identify anti-AD compounds from Chinese herbs. Notably, M308 stands out as a promising anti-AD candidate for development.

## 1. Introduction

With the increasing trend of the aging of the world population, the incidence rate of dementia is increasing, and 152 million people worldwide will suffer from that disease by 2050 [1]. The most common form of dementia is Alzheimer’s disease (AD), accounting for 60–70% of all forms [2]. The typical clinical symptoms of AD are memory and cognitive declines, behavioral disorders, and language impairments. The pathology is characterized by the extracellular senile plaques (SPs) from aggregated β-amyloid (Aβ), intracellular neurofibrillary tangles (NFTs) from hyperphosphorylated Tau protein, and severe neuronal loss [3,4]. Decreased levels of acetylcholine (ACh) and loss of cerebral cholinergic neurons are significant factors causing cognitive dysfunction in AD [5].

So far, the FDA has approved six drugs for AD treatment: Tacrine, Donepezil, Rivastigmine, Galantamine, Memantine, and Aducanumab. Memantine is a glutamate receptor antagonist among the first five drugs; the other four are acetylcholinesterase inhibitors [6]. While more widely used in clinical applications, they are only small molecule drugs for symptom relief. Aducanumab can target Aβ, classified as a small molecule disease modifier for AD. However, whether Aducanumab, which has just been approved for marketing, will slow cognitive decline remains intensely uncertain and controversial [7]. In addition, this drug will be sold for use at ¥360,000 ($56,000) per person per year, which will place a heavy financial burden on society and the patient’s family. Therefore, some emerging strategies for drug discovery are urgently needed to address the current dilemma.

Traditional Chinese Medicines (TCMs) are characterized by fewer side effects, lower prices, and more targets [8,9,10,11]. Especially in long-term clinical practice, TCMs possess more favorable therapeutic value for treating various complex diseases, such as AD [10,12,13]. From the perspective of TCM, blood stasis is deemed an essential causative factor of dementia, so activating blood circulation and removing stasis is one of the critical therapies for AD. Radix Salviae Miltiorrhizae (Danshen), a classic blood-activating and stasis-dispelling medicinal, is listed firstly in Shennong’s Classic of Materia Medica. Modern research has shown that Danshen has a potential protective effect against Alzheimer’s disease [14,15,16,17], but definitive pharmaceutical efficacy substances in anti-AD are still not well recognized.

Recently, the molecular mechanisms of TCMs can also be shed more comprehensively, owing to innovative computational methods and high-throughput technology in drug discovery. For one thing, systematics-based network pharmacology can be leveraged to evaluate the TCM effect as a whole unity [18,19,20,21]. Furthermore, network target has been proposed as a therapeutic concept based on TCM’s holistic theory, which is different from a single target in new drug R&D for Western medicine [22]. For another, gene expression analysis tools are well developed, including establishing and improving the Connectivity Map (CMap). The CMap has constructed a genome-scale cellular signature library that codes transcriptional responses to chemical, genetic, and disease perturbation to link biological systems, genes, and drugs [23,24]. Some reports are currently available on the applications of the CMap platform for novel drug R&D and TCM research [25,26]. The gene expression profile can be queried against the CMap CLUE application for each TCM or component. Then some known compounds with similar reference perturbagen signatures are retrieved and analyzed, reflecting the overall effect of the queried agent on the biological system.

In this work, we proposed a novel strategy for anti-AD drug discovery from TCMs based on network target. we first collected genes associated with AD and putative targets for plasma absorbed compounds of Danshen. Then, following the mapping of putative targets to AD-related genes, molecular docking was employed to confirm the therapeutic targets for AD. Next, a subnetwork of Danshen-treated AD was constructed, along with a multifunctional analysis. Furthermore, a network-based scoring was defined to assess each compound’s anti-AD effect to identify the best anti-AD drug candidates. Meanwhile, the network algorithm was verified by the latest CMap resource to explore the molecular mechanisms using gene expression signatures. Lastly, ex vivo experiments were conducted to verify whether Salvia miltiorrhiza-containing plasma (SMP) possesses the neuroprotective effect via measuring cell viability, ACh levels, anti-apoptosis, and anti-oxidative stress effect. Furtherly, an RT-qPCR assay was performed to demonstrate the anti-AD activity of the candidate component from SMP. The research was expected to identify the most promising anti-AD ingredients from Danshen, contributing to developing more effective and affordable drugs for AD patients.

## 2. Results and Discussion

### 2.1. Target Identification and Validation

Danshen can exert promising therapeutic effects on AD from a network pharmacology perspective. The following three aspects supported this result.

**Figure 1:**
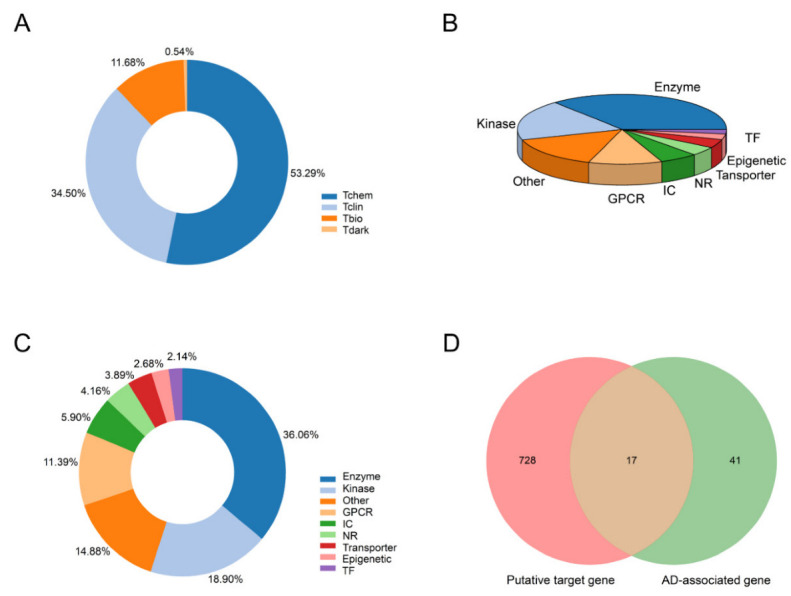
The putative target distribution of (**A**) target development/druggability levels; (**B**) target family in Tclin and (**C**) target family in all; (**D**) targets mapped to AD-related genes.

Firstly, the putative target distribution of Danshen was consistent with existing marketed drugs. Our previous data from the Danshen secondary metabolites database indicated at least 414 ingredients in Danshen. However, only 52 compounds can be detected in plasma (see Table S1). We collected 745 putative targets for those compounds by integrating several databases. Pharos is a comprehensive, integrated knowledge portal for elucidating the uncharacterized or poorly annotated portion of the Druggable Genome. We input each putative target to the Pharos database (Version 3.8.1, https://pharos.ncats.nih.gov, accessed on 13 October 2021) [27] to acquire their target development level and target family. According to Illuminating the Druggable Genome Knowledge Management Center (IDG-KMC), current targets could be categorized into four development/druggability levels: Tclin, Tchem, Tbio, and Tdark. For Tclin, these targets have at least one approved drug with known mechanisms of action. Targets with at least one ChEMBL compound with less than 30 nM activity cutoff value are classified as Tchem. As shown in Figure 1A, Tclin and Tchem account for 34.5% and 53.29% of all putative targets, indicating a high potential for the medicinal effect of Danshen.

Furthermore, drugs often regulate the activity of a variety of proteins. Thus, we also drew the distribution of the target family of all plasma-absorbed Danshen compounds (see Figure 1B,C). The target families were divided into Enzyme, Kinase, G protein-coupled receptor (GPCR), ion channel (IC), nuclear receptor (NR), Transporter, Epigenetic, transcription factor (TF), and others. Enzymes take the highest proportions in Tclin targets and all targets, followed by kinases and GPCRs. By 2015, there were 549 human-genome-derived proteins associated with the therapeutic efficacy of approved small molecule drugs, among which GPCRs, ICs, and kinases occupy a vital position [28]. In particular, GPCR is the most prominent family of membrane receptor proteins in the human body, closely related to human diseases, and targets more than 40% of marketed drugs [29].

Secondly, the verified targets by molecular docking exhibited clinical applicability for anti-AD. We then applied rigorous filtering conditions such as the Elite gene to the MalaCards database to obtain the most directly associated disease genes. In sum, 58 AD-related genes were screened out as disease dataset (see Table S2). Next, we mapped the targets of the components to the AD-related diseases by intersection analysis, and 17 potential target genes were obtained, as shown in Figure 1D.

**Figure 2:**
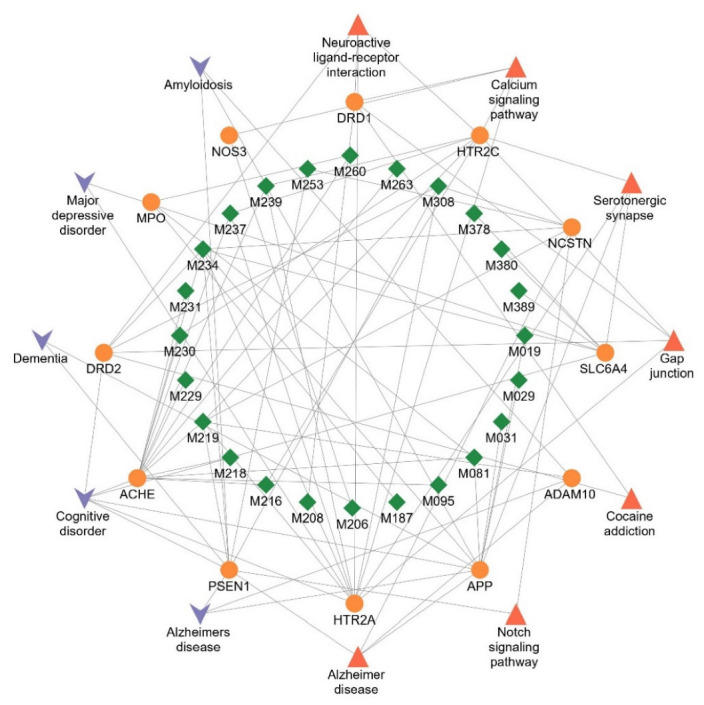
The compound-target-pathway/disease network of Danshen. Green diamonds denote compounds verified by molecular docking. Orange circles refer to validated targets for Danshen. Red triangles denote pathways enriched in KEGG (*FDR* < 0.05). Purple V-shapes represent diseases enriched in DISEASE (*FDR* < 0.05).

Through in silico docking, four genes were eliminated due to their low binding affinity. As shown in Table 1, 25 active ingredients were confirmed to bind with 13 target proteins related to AD. The verified targets were NCSTN, APP, PLAU, MPO, NOS3, PSEN1, ADAM10, ACHE, HTR2A, DRD2, HTR2C, SLC6A4, and DRD1. Except for compound M001 and target PLAU, the compound-target-pathway/disease interaction was shown in Figure 2, suggesting Danshen can exert therapeutic effects on AD by these targets. According to the Therapeutic target database [30], ACHE and DRD2 belong to Successful targets, corresponding to at least one approved drug. There are at least five FDA-approved drugs for AD (Donepezil, Galantamine, Huperzine A, Rivastigmine, Tacrine) as Acetylcholinesterase inhibitors, targeting ACHE to raise ACh levels [31]. DRD2 is a therapeutic target of Dihydroergotoxine (Vasodilator Agents for Dementia) [32] and Memantine (Glutamate receptor antagonist for moderate to severe Alzheimer’s type Dementia) [33].

**Figure 3:**
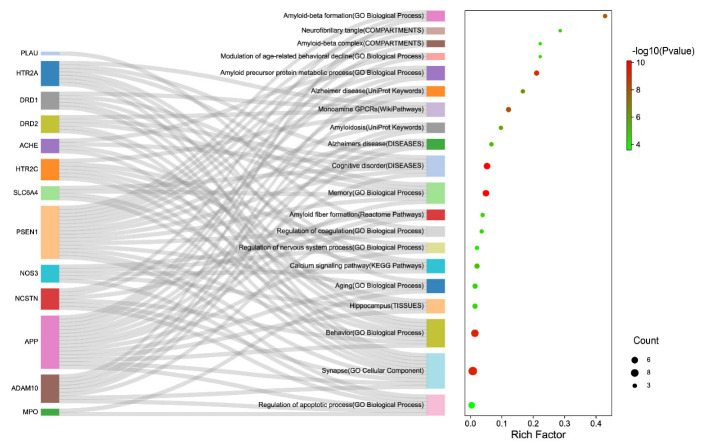
The multi-dimensional enrichment analysis based on validated targets, including Gene Ontology, KEGG Pathways, Reactome Pathways, WikiPathways, Tissue expression (TISSUES), Subcellular localization (COMPARTMENTS), and Annotated Keywords (UniProt).

Further, the multi-dimensional enrichment analysis based on validated targets was accomplished. Figure 3 showed that APP and PSEN1 occurred most frequently in multiple enrichment entries. Aβ is generated from amyloid precursor protein (APP, gene synonym: AD1), whose targeting amyloidogenic processing is a critically plausible strategy for developing disease-modifying therapies [34]. PSEN1 (synonym: AD3), corresponding to presenilin-1 protein, as the catalytic subunit of the gamma-secretase complex, is involved in intramembrane cleavage of integral membrane proteins such as Notch receptors and APP [35].

Finally, the verified targets were involved in multiple AD pathological mechanisms. As shown in Figure 3, these targets were significantly enriched in the hippocampus (BTO:0000601), memory and behavior (GO:0007613, GO:0007610), regulation of neurological processes (GO:0031644), modulation of age-related behavioral decline (GO:0090647), cognitive disorder (DOID:1561), suggesting that these targets participate in the regulation of the central nervous system. Moreover, these were significantly enriched to Alzheimer’s disease (DOID:10652, KW-0026). Furthermore, based on multiple enrichment results, AD treatment with Danshen involved several mechanisms, including Aβ, NFTs, and neurovascular repairment, reflecting the holistic approach to treating disease in TCM. The most enriched was related to Aβ, including amyloidosis (KW-1008), amyloid fiber formation (HSA-977225), amyloid-beta complex (GOCC:0106003), amyloid-beta formation (GO:0034205), amyloid precursor protein metabolic process (GO:0042982), and others.

Next, NFT (GOCC:0097418), synapse (GO:0045202), and regulation of apoptotic process (GO:0042981) were found together, which illustrated that the premature death of cholinergic neurons might be associated with the accumulation of neurofibrillary tangles [36]. Meanwhile, the calcium signaling pathway (hsa04020) was also significantly enriched, where calcium disruption is ubiquitously involved in all AD pathologies [37]. In addition, Danshen is a Chinese herb that activates blood circulation and participates in the regulation of coagulation (GO:0050818), which could also serve as evidence regarding neurovascular repairment of cerebral amyloid angiopathy (CAA) in AD patients [38]. Aβ possesses neurotoxicity and provokes amyloidosis of the vascular wall, leading to sclerosis, poor elasticity, and even a tendency to rupture or thrombosis. The neurotoxicity can also trigger premature apoptosis of the neuronal cells. Thus, apoptosis can occur in Aβ, NFT, and CCA. The anti-AD effect of Danshen is likely related to the inhibition of neuronal apoptosis.

### 2.2. Network Construction and Analysis

**Figure 4:**
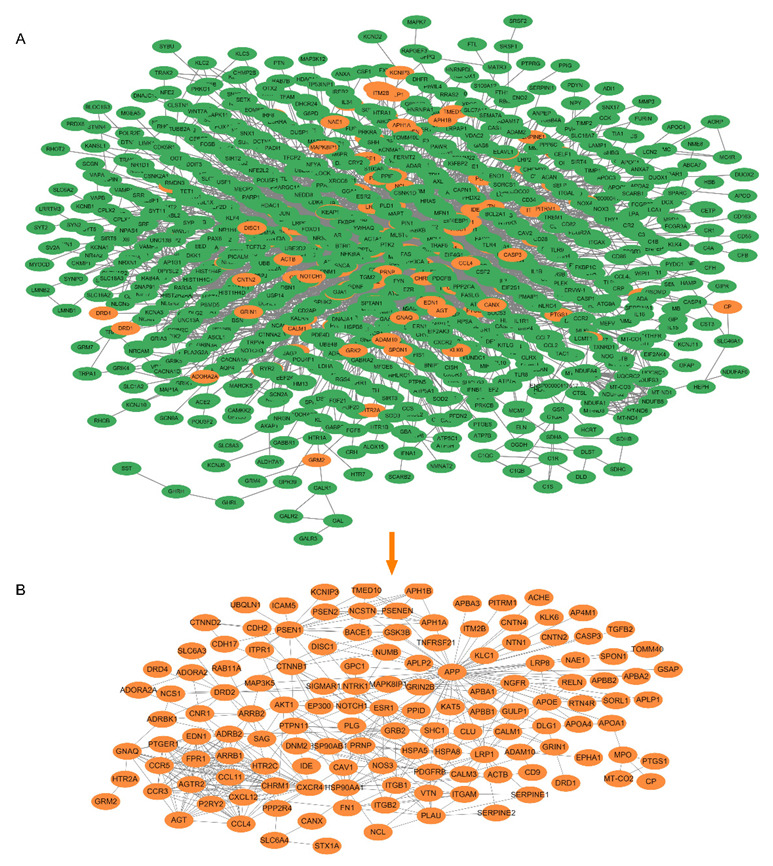
Subnetwork construction. (**A**) AD background network and (**B**) DS-AD network (subnetwork for Danshen-treated).

Due to the complex etiology of Alzheimer’s disease, achieving satisfactory outcomes with a single target or a combination of several targets as a therapeutic strategy is challenging. The advantages and potential of TCM and its active ingredients for treating Alzheimer’s disease lie in that TCM emphasizes a holistic outlook in treating the disease. Therefore, we study Danshen on Alzheimer’s disease from the perspective of network targets. To begin with, we required a high-quality PPI background network, a biological network model of Alzheimer’s disease. Text mining was applied to generate a physical AD network, which is relatively more reliable than a functional (predictive) network. Hence, the constructed network consisted of 1387 nodes and 6082 directed links, where the nodes represent proteins, and the links denote their relationships (see Figure 4A). Next, the subnetwork of the Danshen action was extracted. The validated targets by molecular docking served as core nodes. Since the importance of a node in the network is associated with its information and the neighboring nodes, we also consider the neighbors of the 13 core nodes. As such, the DS-AD network contained 136 nodes and 421 links. The results are presented in Figure 4B.

**Figure 5:**
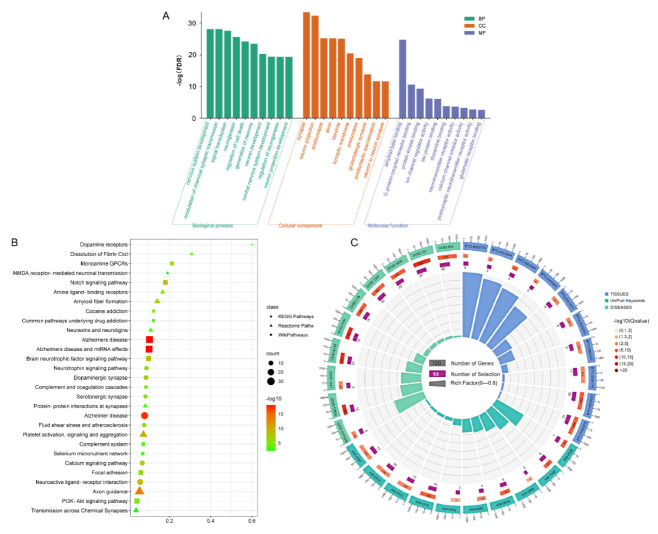
The multi-dimensional enrichment analysis based on the DS-AD network, where (**A**) GO analysis; (**B**) pathway analysis; (**C**) other analysis, including TISSUES, DISEASES, and UniProt Keywords.

The strong association of this subnetwork with AD was demonstrated by enrichment analysis of the 134 nodes encompassed by the DS-AD network. As shown in Figure 5A, GO analysis showed that nervous system development (GO:0007399), modulation of chemical synaptic transmission (GO:0050804) for GO Biological Process; synapse (GO:0045202), neuron projection (GO:0043005) for GO Cellular Component; amyloid-beta binding (GO:0001540), tau protein binding (GO:0048156) for GO Molecular Function, etc. Some pathways were also significantly enriched as follows (Figure 5B): KEGG Pathways such as Alzheimer’s disease (hsa05010), neuroactive ligand-receptor interaction (hsa04080); Reactome Pathways such as axon guidance (HSA-422475), amyloid fiber formation (HSA-977225); WikiPathways such as Alzheimer’s disease (WP5124), Notch signaling pathway (WP61). In Figure 5C, other enrichment analyses included diseases, tissues, and keywords. Amyloidosis (DOID:9120), Alzheimer’s disease (DOID:10652), and central nervous system disease (DOID:331) were enriched in DISEASES; Cerebral cortex (BTO:0000233), Alzheimer’s disease-specific cell type (BTO:0000590) in TISSUES; and amyloidosis (KW-1008), Notch signaling pathway (KW-0914), and Alzheimer’s disease (KW-0026) in UniProt Keywords. The Notch signaling pathway is a cellular signaling system involved in vascular development and function. A relationship has been reported between altered Notch signaling and the pathophysiology of Alzheimer’s disease [39].

It was found that the enrichment results calculated by different dimensions and different backgrounds were mainly in agreement with each other. The established subnetwork of Danshen efficacy involves several aspects, primarily concerning amyloidosis and cerebral neurovascular disorders, consistent with Cao et al. [40]. More importantly, DS-AD network enrichment analysis revealed that the mechanism of treating AD by Danshen was also associated with anti-apoptosis, which can be backed up by the following: regulation of cell death (GO:0010941), calcium channel inhibitor activity (GO:0019855), Apoptosis (KW-0053), Neurotrophin signaling pathway (hsa04722), Calcium signaling pathway (hsa04020), PI3K-Akt signaling pathway (WP4172).

### 2.3. Anti-AD Effect Analysis of Danshen Component

#### 2.3.1. Score Ranking

To determine which components were more active against AD among 25 active ingredients of Danshen, we used a model network to assess the relative strength of each ingredient’s effect. The algorithm integrated network topology and propagation dynamics kinetics [41]. The compounds ranked based on the DS-AD network for their action activity, as shown in Table 2. It was reflected that the effect strength of different components on AD varies considerably, with a four-order-of-magnitude difference between the highest score (0.647295) and the lowest score (4.0 × 10^−5^).

In addition, to investigate if components indicated a significant anti-AD effect, an equal number of target proteins were randomly generated for each component. The mean effect score and the standard deviation of the 1000 random counterparts were calculated. Hence the Z-scores of the anti-AD effect for compounds were obtained, listed in Table 2. The Z-scores of M234 (1R-hydroxymiltirone, 105037-82-9) and M308 (Danshenxinkun D, 98873-76-8) are 5.798866 and 4.93371, which are greater than 3, implying a significant anti-AD effect of these single compounds.

We further compare the anti-AD effect of the compound with those of FDA-approved drugs (Donepezil and Memantine) for Alzheimer’s disease. Similarly, the network score and Z-score for the anti-AD effect of each drug were calculated. The Z-score is 2.28841 and 2.569619, all of them are bigger than 1.96, suggesting that two drugs significantly affect AD (*p* < 0.05). As an acetylcholinesterase inhibitor, Donepezil treats mild to moderately severe AD patients. The targets of Donepezil are ACHE and HTR2A, which were included in our docked results. Memantine is a drug for the treatment of moderate to severe AD. The pharmacological classification of Memantine belongs to NMDA receptor antagonists. The reported targets are CHRFAM7A, CYP2E1, DRD2, GRIN1, GRIN2A, GRIN2B, GRIN3A, and HTR3A. Among those, DRD2 also is one of our verified targets.

The network scores did not necessarily represent the actual biological activity of the components but can be utilized roughly to indicate the relative strength of each component. We checked the activity value of both two compounds through Drugcentral. The average IC50:5.386 (−log[M]) for donepezil and 4.95 (−log[M]) for Memantine indicated that the activity of Donepezil was weaker than that of Memantine to some extent, which was consistent with the scoring result. The anti-AD scores of M234 and M308 were greater than those of the positive drugs, so both were considered superior to the two current classes of drugs in effect.

#### 2.3.2. CMap Validation

Tanshinone IIA and Danshensu have been predominantly reported to exhibit anti-AD activity [16,42]. However, the activity of both in our research was significantly weaker than that of M234 and M308. To demonstrate the reliability of our findings based on the network target, an analytical approach that can mirror the overall landscape is necessary, which is expected to illuminate the effect of a component on the biological network. So, we performed a comparative analysis between the expression signatures in response to compound perturbations via Connectivity Map. Since the transcriptome data generated by perturbation from M234 and M308 were not readily available, we only compared the pharmacological mechanisms between these two compounds (Tanshinone IIA and Danshensu) and the positive control (Donepezil and Memantine).

**Figure 6:**
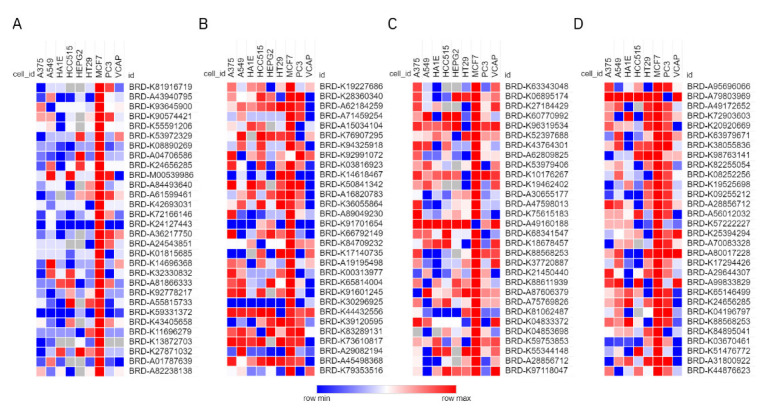
The heat map of connectivity scores for (**A**) Danshensu, (**B**) Tanshinone IIA, (**C**) Donepezil, and (**D**) Memantine.

In the results of the CMap analysis, the top 30 instances of positive correlations were presented in Figure 6. The outcome from the MCF-7 cell line exhibited a significant difference compared to the perturbation that acted on the other eight core cell lines. Therefore, only the similarity of gene expression in MCF-7 was focused on. The expression signatures of Donepezil were highly positively correlated with Pergolide (BRD-K60770992), and Memantine was highly positively correlated with Propofol (BRD-K82255054) in the top 10 ranked compound perturbations. Their ATC codes (N04BC02 and N01AX10) exert medicinal effects on the nervous system (see Table S3). However, for Tanshinone IIA and Danshensu, we did not find any drugs with high positively similar specific action on the nervous system. Furthermore, the results revealed that Tanshinone IIA and Danshensu are highly correlated with cardiovascular system drugs, such as Cymarin (BRD-A71459254, C01AC03), Bucladesine (BRD-A04706586, C01CE04). Indeed, Tanshinone IIA has been successfully developed and marketed as a drug, Sulfotanshinone sodium injection (WS-10001-(HD-1014)-2002), for the adjuvant treatment of coronary heart disease, angina pectoris, and myocardial infarction, which agrees with the results of Tanshinone IIA for CMap analysis. So, it is evidence that the anti-AD activity of Tanshinone IIA and Danshensu is weaker than that of Memantine and Donepezil just from the perspective of drug action positioning, further supporting the outcomes of the network scoring.

#### 2.3.3. Anti-AD Drug Candidates from Danshen

It was clear from the above that M234 (1R-hydroxymiltirone) and M308 (Danshenxinkun D) showed relatively strong anti-AD activity among all components of Danshen. The SMILES of two compounds were input to the SwissADME (http://www.swissadme.ch/, accessed on 15 October 2021) [43], and a BOILED-Egg statistical model was applied to predict blood-brain barrier (BBB) penetration [44]. It was seen that they could both cross the BBB, which is fundamental for the distribution of central-acting drugs.

**Figure 7:**
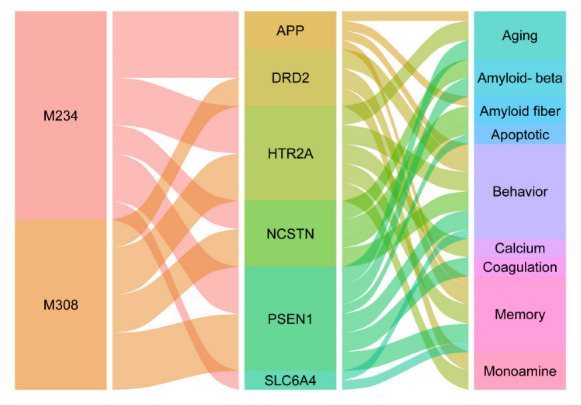
The alluvial plot of compound-target-pathway for M234 and M308.

As shown in Figure 7, these two have the common feature of multiple targets, seven targets for M234 and four targets for M308. To some extent, this reflected the excellence of both for the network target (DS-AD network). Moreover, the pathway analysis also showed that they were both involved in regulating the central nervous system, which can improve AD symptoms. In addition, they both participate in regulating the neuronal apoptotic process, thereby protecting the nervous system.

### 2.4. Experimental Evaluation of Neuroprotective Effects

The above analysis shows that both Danshen and M308 can exert neuroprotective effects through anti-apoptosis. Remarkably, the PI3K-Akt signaling pathway (WP4172), Calcium signaling pathway (hsa04020), and Neurotrophin signaling pathway (hsa04722) were involved in the DS-AD network (Figure 5). Therefore, we then performed ex vivo experiments to validate the anti-apoptotic and anti-oxidative stress effects of SMP. The candidate anti-AD component M308 was shown to be present in SMP by the MS method, suggesting that M308 is involved in the neuroprotective effect of SMP. Subsequently, the expression of target genes in the DS-AD network by M308 was further analyzed, providing evidence of a neuroprotective effect for M308. PC12 cells, a rat pheochromocytoma-derived cell line with good neuronal properties, are commonly used as nerve cell models to study the molecular protective mechanisms of some candidate drugs against neurological diseases, such as AD [45]. Therefore, PC12 cells were selected for the present study.

#### 2.4.1. SMP Protects against H_2_O_2_-Treated PC12 Cells

**Figure 8:**
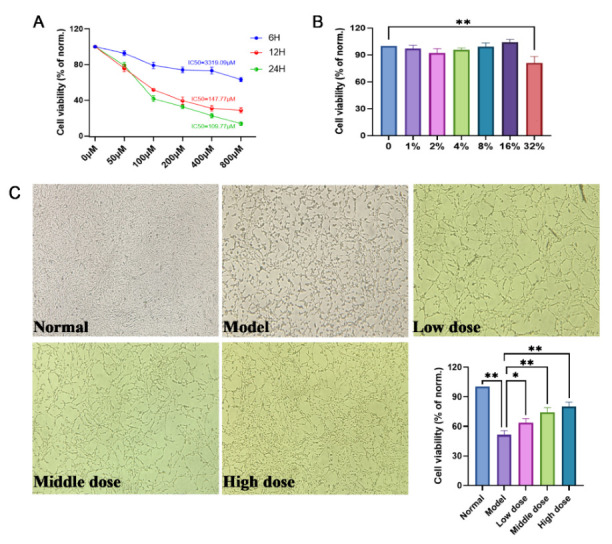
Protective effects of SMP on H_2_O_2_-induced PC12 cells. (**A**) Effects of various concentrations and treatment times of H_2_O_2_ on the viability of PC12 cells; (**B**) Effects of SMP on the viability of normal PC12 cells; (**C**) Effects of SMP (4%, 8%, and 16%) on the viability of H_2_O_2_-induced PC12 cells. SMP: Salvia miltiorrhiza-containing plasma. For data expressed as mean ± SD (*n* = 3), * *p* < 0.05 and ** *p* < 0.01 vs. model group.

Figure 8A illustrates the effects of various concentrations of H_2_O_2_ on the viability of PC12 cells at different times. According to the experimental results, we choose 100 μM as the best working concentration of H_2_O_2_ and the optimal working time of 24 h (IC_50_ =109.77 μM). As shown in Figure 8B, when the concentration of SMP was less than 16%, the viability of PC12 cells was over 90%. However, over 32%, the viability of PC12 cells was greatly affected (*p* < 0.01). Therefore, we selected the SMP at concentrations of 4%, 8%, and 16% as the working concentrations to further investigate the possible molecular action mechanisms of SMP. Moreover, it can be seen from Figure 8C that SMP could increase the cell viability of H_2_O_2_-stimulated PC12 cells compared with the model group in a dose-dependent manner. These results indicated that SMP inhibited H_2_O_2_-treated PC12 cell injury.

#### 2.4.2. SMP Increased Ach Levels in H_2_O_2_-Stimulated PC12 Cells

ACh is a major neurotransmitter in the brain, and the decreased level contributes to memory loss [5,46]. As shown in Table 3, H_2_O_2_ administration significantly reduced Ach content within the cells, and SMP (8% and 16%) significantly increased ACh levels in a dose-dependent manner in PC12 cells. This study showed that Danshen could improve memory in AD patients by enhancing the level of acetylcholine.

#### 2.4.3. SMP Suppresses Apoptosis in H_2_O_2_-stimulated PC12 Cells

**Figure 9:**
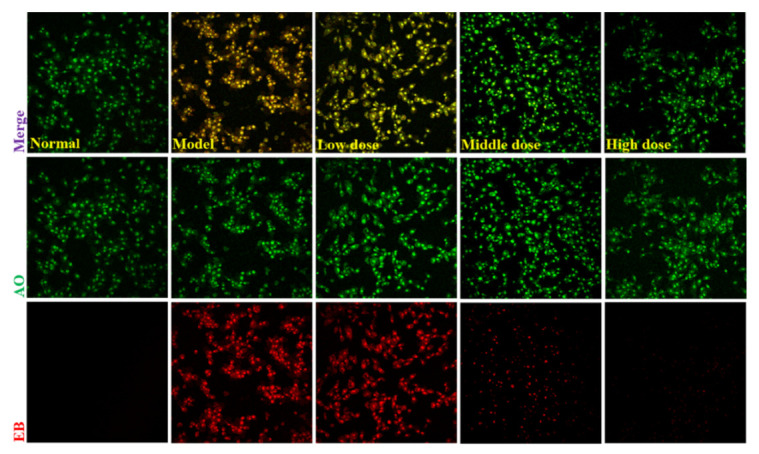
Apoptotic assay by AO-EB staining (×20). Cells were pretreated with SMP (4%, 8%, and 16%) for 24 h and then incubated in the presence of H_2_O_2_ (100 μM) for 24 h. SMP: Salvia miltiorrhiza-containing plasma. The results were observed using laser confocal microscopy under a 20× microscope.

AO/EB double staining is an ideal approach for evaluating the nuclear morphological changes of apoptotic cells. AO can penetrate cells with intact cytomembrane and emit bright green fluorescence. In contrast, EB can only penetrate cells with damaged cytomembrane and release bright orange/red fluorescence [47]. In this way, AO/EB staining displays green, yellow, and orange/red to represent living, early apoptotic, and late apoptotic cells [48]. To further confirm whether Danshen (SMP) can suppress apoptosis or not, AO/EB staining was carried out by laser confocal microscope. As indicated in Figure 9, cells in the normal group were almost uniformly green with normal and homogeneous nuclei. Whereas cells in the model group were in irregular shape, instead, karyopyknosis were observed (stained bright yellow-orange), which indicates that H_2_O_2_ can induce apoptosis in PC12 cells. Additionally, the yellow-orange apoptotic cells were significantly reduced, and the normal morphology of green cells was significantly increased after SMP administration from low to high concentrations. Our results suggest that SMP can significantly inhibit apoptosis in PC12 cells treated with H_2_O_2_.

**Figure 10:**
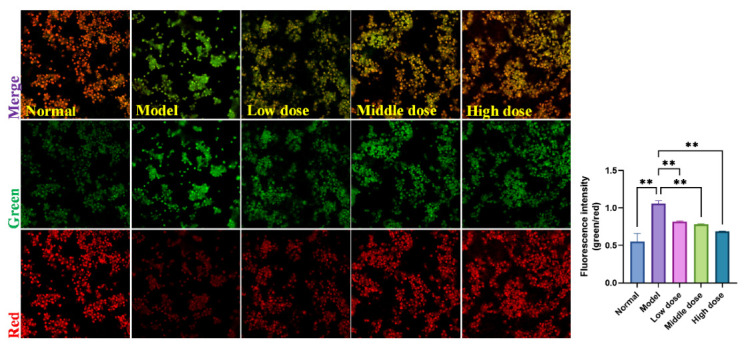
Effects of SMP on the ΔΨm in PC12 cells (×20). Cells were pretreated with SMP (4%, 8%, and 16%) for 24 h and then incubated in the presence of H_2_O_2_ (100 μM) for 24 h. ΔΨm was measured using a JC-1 assay kit and observed using laser confocal microscopy under a 20× microscope. SMP: Salvia miltiorrhiza-containing plasma. The values were represented as the mean ± SD (*n* = 3). ** *p* < 0.01 vs. model group.

Furthermore, we quantitatively examined the effects of SMP on cell apoptosis by measuring mitochondrial membrane potential (MMP, *ΔΨ*m, green/red fluorescence ratio), whose decline is considered an early characteristic of apoptosis cells. We used the JC-1 probe to detect the changes of *ΔΨ*m in PC12 cells exposed to H_2_O_2_. Results of the *ΔΨ*m determination are represented in Figure 10. After being incubated with H_2_O_2_ for 24 h, the red fluorescence in PC12 cells significantly decreased, and the corresponding green fluorescence increased, indicating the decline in MMP. However, there was less reduction in red fluorescence in the SMP-pretreated cells than in the model group, suggesting that SMP could reverse H_2_O_2_-induced MMP decline.

#### 2.4.4. SMP Reduced the H_2_O_2_-Stimulated Reactive Oxygen Species (ROS) Generation in PC12 Cells

**Figure 11:**
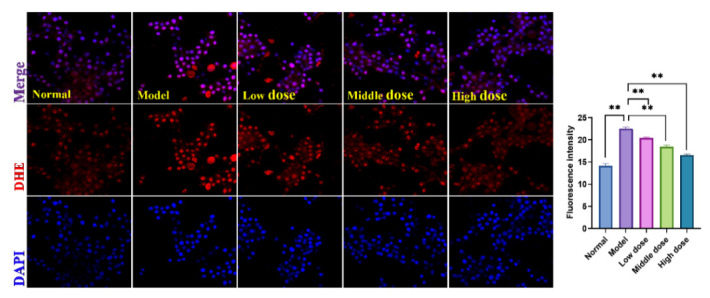
Effects of SMP on ROS levels in H_2_O_2_-induced PC12 cells (×40). PC12 cells were treated with SMP (4%, 8%, and 16%) for 24 h and subsequently subjected to H_2_O_2_ (100 μM) for 24 h. The intracellular ROS levels in the PC12 cells were observed by laser confocal microscopy under a 40× microscope. SMP: Salvia miltiorrhiza-containing plasma; ROS: reactive oxygen species. The values were expressed as mean ± SD (*n* = 3), ** *p* < 0.01 vs. model group.

The above findings show that SMP can suppress H_2_O_2_-stimulated apoptosis in PC12 cells. In addition, cells stimulated by H_2_O_2_ will produce excessive ROS, which is also a significant cause of cell apoptosis [49]. We used the DHE probe to determine the effect of SMP treatment on ROS levels in H_2_O_2_-stimulated PC12 cells. As shown in Figure 11, H_2_O_2_ stimulation significantly increased ROS levels in PC12 cells compared with the normal group. More importantly, SMP pretreatment could inhibit ROS levels in H_2_O_2_-treated PC12 cells.

#### 2.4.5. SMP Ameliorated H_2_O_2_-Induced Oxidative Stress in PC12 Cells

**Figure 12:**
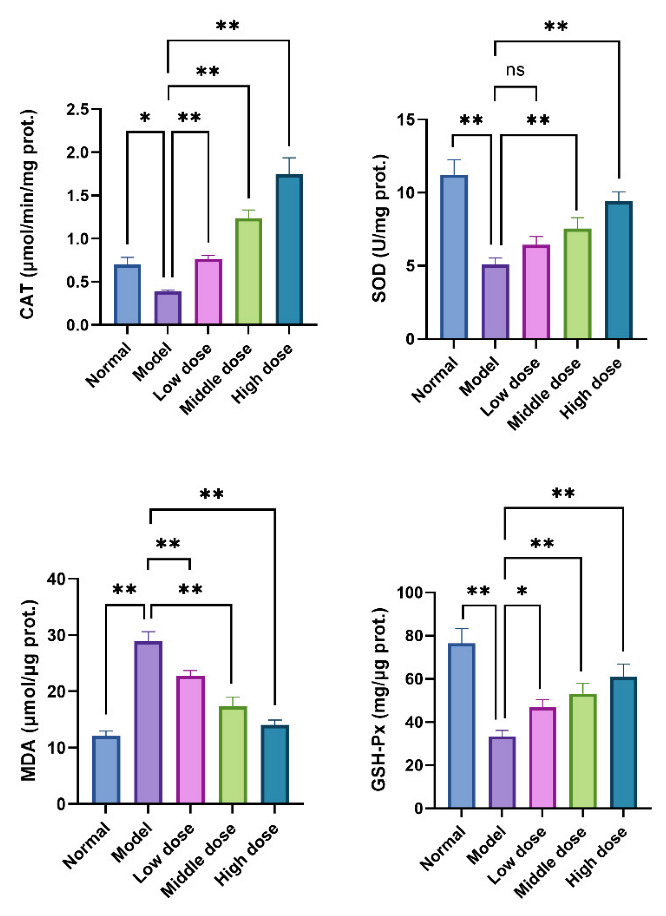
Effects of SMP on CAT, SOD, GSH-Px, and MDA in H_2_O_2_-stimulated PC12 cells. The levels of MDA and activities of CAT, SOD, and GSH-Px were determined by commercial assay kits. PC12 cells were treated with SMP (4%, 8%, and 16%) for 24 h and subsequently subjected to H_2_O_2_ (100 μM) for 24 h. SMP: Salvia miltiorrhiza-containing plasma; CAT, catalase; SOD: superoxide dismutase; MDA: malondialdehyde; GSH-Px: glutathione peroxidase. The values were represented as the mean ± SD (*n* = 3). ns: no significant difference, * *p* < 0.05 and ** *p* < 0.01 vs. model group.

Intracellular CAT, SOD, GSH-Px, and MDA are commonly used biomarkers to assess the oxidative stress level of cells or tissues. When cells suffer from oxidative stress, their intracellular antioxidant enzyme system will be activated to inhibit the excessive production of ROS. CAT, SOD, and GSH-Px are cells’ most critical scavenging enzymes of reactive oxygen species. In addition, the oxidation product of lipid peroxidation caused by oxidative stress is MDA, which is cytotoxic. The detection of MDA levels also allows the assessment of intracellular levels of oxidative stress. Therefore, to evaluate the effect of SMP on H_2_O_2_-induced oxidative stress, we also examined the generation of MDA and the activities of CAT, SOD, and GSH-Px in PC12 cells. The results are shown in Figure 12, the MDA level in PC12 cells was increased significantly after 24 h H_2_O_2_ stimulation, compared with the normal group. The pretreatment with SMP could inhibit MDA production in a concentration-dependent manner. In addition, H_2_O_2_ stimulation can reduce the activities of antioxidant enzymes CAT, GSH-PX, and SOD in cells. However, SMP pretreatment can enhance the actions of these enzymes. These results suggest that SMP can protect PC12 cells from oxidative damage caused by H_2_O_2_ by increasing the activity of ROS scavenging enzymes.

#### 2.4.6. Effects of M308 and SMP on mRNA Expressions of PSEN1, DRD2, and APP in H_2_O_2_-Stimulated PC12 Cells

M308 (Danshenxinkun D) was an anti-AD candidate for Danshen based on the efficacy score of DS-AD network targets, and the reliability of the screening strategy was demonstrated by in silico CMap analysis. Further, a classical natural drug discovery approach by pharmacological activity tracing was then used to support the anti-dementia effect of M308. In previous experiments, Danshen (SMP) has been shown to increase acetylcholine levels, anti-apoptosis, and anti-oxidative stress, thus exerting an anti-AD effect. Among the active components comprising SMP, we focused on whether M308 is present and whether M308 exerts the same effects as SMP at the gene expression level.

**Figure 13:**
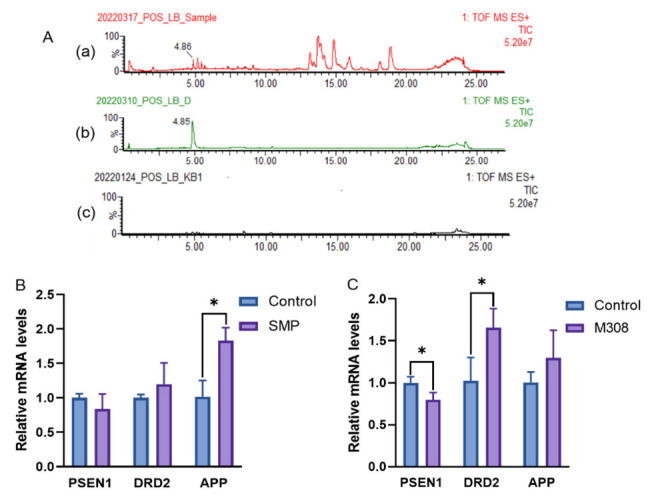
Effects of SMP (16%) and M308 (100 μg/mL) on PSEN1, DRD2, and APP mRNA expression in the H_2_O_2_-treated PC12 cells. (**A**): the TIC of SMP (**a**), M308 (**b**), and blank plasm (**c**) under positive mode; the relative mRNA levels of PSEN1, DRD2, and APP under treatment of SMP (**B**) and M308 (**C**). SMP: Salvia miltiorrhiza-containing plasma; M308: Danshenxinkun D. The gene expressions were normalized to GAPDH and analyzed using the 2^−ΔΔCT^ method. The Data were represented as the mean ± SD *(n* = 3). * *p* < 0.05 vs. the control group.

As shown in Figure 13A, M308 was detected in SMP under the positive ion mode of mass spectrometry and identified by the reference compound, suggesting that M308 may participate in the anti-AD effect of SMP. Further analysis by RT-qPCR showed that the effects of M308 and SMP on mRNA expressions of PSEN1, DRD2, and APP were consistent. The mRNA expressions of DRD2 and APP were upregulated, whereas PSEN1 was downregulated compared with those in the control group. The tendency of the PSEN1 and APP mRNA expression levels was consistent with the literature [50]. Through preliminary molecular docking, PSEN1, DRD2, and APP were confirmed to be associated with the anti-AD efficacy of Danshen. As shown in Figure 13B, SMP treatment (16%) influenced the expression of all three genes, especially APP. SMP treatment significantly upregulated (*p* < 0.05) mRNA expressions of APP compared with those in the control group. In addition, the successful docking of M308 with PSEN1 and DRD2 was further verified in this experiment. Figure 13C displayed that the PSEN1 mRNA expressions were significantly decreased (*p* < 0.05) in M308-treated (100 μg/mL) group, while the DRD2 expression was increased (*p* < 0.05). As mentioned above, DRD2 and PSEN1 are vital in treating AD. It has been reported that PSEN1 mRNA expression was increased in AD patients, while DRD2 mRNA and APP mRNA was decreased [51,52,53]. In the present study, M308 could significantly reverse the expression of PSEN1 and DRD2 in H_2_O_2_-treated PC12 cells. Moreover, because PSEN1 is associated with APP [38], M308 may indirectly affect the change of APP mRNA expression as SMP, although there is no significant difference. All these further prove the anti-AD effect of M308 in SMP.

## 3. Materials and Methods

### 3.1. Data Mining

#### 3.1.1. AD-Associated Genes Screening

The AD-associated genes were found in DisGeNET gene-disease association database (http://www.disgenet.org/ accessed on 18 September 2021) and MalaCards human disease database (http://www.malacards.org/ accessed on 18 September 2021). The CURATED and DSI-g > 0.65 and Elite genes were selected as the retrieval parameter in DisGeNET and MalaCards, respectively. In addition, the relevant therapeutic target genes were queried in the Drugbank 3.0 database (https://www.drugbank.ca/ accessed on 18 September 2021, Pharmacological action: yes). The genes selected in the three databases were merged, and the duplicates were removed.

#### 3.1.2. Danshen Compounds Absorbed in Plasma and Their Putative Targets

Together with our previous study and literature reports [54,55], active components absorbed in plasma were collected. Data about target proteins for those compounds were accessed from following resources: ETCM database (http://www.tcmip.cn/ETCM/ accessed on 20 September 2021, yes), STITCH database 5.0 (http://stitch.embl.de/ accessed on 20 September 2021, Score > 0.7), Swiss target prediction database (http://swisstargetprediction.ch/ accessed on 21 September 2021, Probability > 0), SEA database (http://sea.bkslab.org/ accessed on 21 September 2021, HUMAN) or Targetnet database (http://targetnet.scbdd.com/ accessed on 21 September 2021, Probability > 0.5). The predicted targets were integrated for each compound, and the target names were uniformly transformed into corresponding gene names for subsequent bioinformatics analysis.

### 3.2. In Silico Target Validation by Molecular Docking

#### 3.2.1. Intersectional Analysis

The proteins encoded by disease-related genes may be potential targets for therapeutic agents. We investigated the overlaps between AD-associated genes and Danshen putative target genes to seek out potential therapeutic targets of active compounds for AD. The two datasets were uploaded to the OmicShare cloud platform (http://www.Omicshare.com accessed on 23 September 2021). The Venn diagram tool analyzed the common genes as potential efficacy targets.

#### 3.2.2. Molecular Docking

The crystal complexes of potential targets from Homo sapiens were downloaded from the Swiss Prot/Uniprot protein sequence database (https://www.uniprot.org/ accessed on 25 September 2021) or RSCB protein structure database (PDB, http://www.rcsb.org accessed on 25 September 2021) until September 2020. The files of the target protein, its ligand in situ, and the compounds were transformed into pdbqt format files by Autodocktool software. AutoDock Vina was used to perform batch docking, and relevant parameters are shown in Table S4. When the energy was lower than −5.0 kCal/mol [56,57] or the binding energy of ligands in situ, the ligands and protein receptors were considered highly binding.

### 3.3. Construction of AD Network Treated by Danshen

#### 3.3.1. Construction of AD Background Network

STRING network with AD-associated proteins was loaded based on text mining by Cytoscape StringApp (Version 1.7.0, UCPH, Copenhagen, Denmark) [58]. In order to construct the AD background network with high confidence edges, only a physical subnetwork in Alzheimer’s disease (DOID:10652) was elected. We filtered the network with a score cutoff of 0.9 within 2000 proteins and then checked whether the verified target proteins were covered. If not, we lowered the threshold until all the verified target proteins were included in the AD background network.

#### 3.3.2. Extraction of the Danshen-Treated Subnetwork

It was reported that disease genes are likely to be located in the same network neighborhood as ones in the human PPI network [59]. The verified target proteins and their first neighbors were selected in the AD background network to construct a new network, and the self-loops were removed. Therefore, the subnetwork treated by Danshen was extracted from the AD background network, which is called the DS-AD network.

#### 3.3.3. Function Enrichment Analysis

The multiple function enrichment analyses of the DS-AD network were carried out by using the String database (Version: 11.5, SIB, Lausanne, Switzerland,) and Cytoscape StringApp (Version 1.7.0, UCPH, Copenhagen, Denmark), including Biological Process (Gene Ontology, Bethesda, MD, USA), Molecular Function (Gene Ontology, Bethesda, MD, USA), Cellular Component (Gene Ontology, Bethesda, MD, USA), Reference publications (PubMed, Bethesda, MD, USA), KEGG Pathways, Reactome Pathways, WikiPathways, Tissue expression (TISSUES, Copenhagen, Denmark), Subcellular localization (COMPARTMENTS, Copenhagen, Denmark) and Annotated Keywords (UniProt, Hinxton, Cambridge, UK).

### 3.4. Anti-AD Effect Ranking of Active Ingredient

#### 3.4.1. Network Scoring of Anti-AD Effects of Compounds

Identifying compounds with relatively strong anti-AD activity in Danshen can facilitate the development process of drug discovery. According to the literature [60], a computational method was improved to evaluate compounds for treating AD. The network score of the corresponding ingredient was calculated, and the formula was as follows:

(1) $$\mathrm{Score}=\overset{n}{\underset{i=1}{\sum }}\frac{\mathrm{lg}\left(d_{i}\right)}{\mathrm{lg}\left(d_{max}\right)}\times H_{i}$$

where *d_i_* is the degree value of a single node, *d_max_* is the maximum degree value in the DS-AD network; Since the log10 of 1 is 0, we replaced the 0 of the nodes with 0.1. *H_i_* is the diffusion output heat of a single node in the DS-AD network.

Network propagation is an essential and widely-used algorithm with systems biology applications for disease gene prioritization and protein function prediction [61]. The spread of heat diffusion is controlled by the time parameter t (0.1) via the Cytoscape Diffusion app (version 1.6.1, Trey Ideker Lab, San Diego, CA, USA). A selected set of nodes acted as heat sources (seed node) with the same initial heat (1.0). In addition, the centrality (degree) of a protein was analyzed by the plugin NetworkAnalyzer 3.3.2.

#### 3.4.2. Z-Score

Here, Z-score was applied to quantify the difference between the anti-AD affect scores of a Danshen metabolite and its random equivalents [20]. A higher absolute value of the Z-score suggests a more significant difference.

#### 3.4.3. FDA-Approved Anti-AD Drugs

We searched the Therapeutic target database (TTD, http://db.idrblab.net/ttd/ accessed on 14 October 2021) with the keyword “Alzheimer’s disease”. The targets of drugs are used clinically to treat AD as seed nodes in the DS-AD network to perform heat propagation algorithms. Moreover, we selected two common FDA-approved Anti-AD drugs, Memantine and Donepezil, to compare their anti-AD scores with the ingredients. The data of two drugs, including targets and bioassay, were downloaded from the DrugCentral database (https://drugcentral.org/ accessed on 14 October 2021) [62], which was updated in October 2021.

### 3.5. Network Scores Validated by CMap Analysis

Based on the available data, we selected two constituents from Danshen (Tanshinone IIA and Danshensu) and two FDA-approved drugs (Memantine and Donepezil) for analysis by comparing their differential expression profiles.

#### 3.5.1. Differentially Expressed Genes

The gene expression profiles of Tanshinone IIA and Danshensu are available through the Gene Expression Omnibus (GEO), and GSE85871 was selected [63]. Each group was compared with a control group using GEO2R to identify differentially expressed genes. FDR < 0.1, |log| > 1.

#### 3.5.2. Connectivity Map

CLUE is a cloud-based software platform for analyzing perturbation datasets generated using gene expression (L1000) and proteomics (P100 and GCP) assays. In order to retrieve the connected signatures, the upregulated and downregulated genes for Tanshinone IIA and Danshensu were queried against the CLUE platform (https://clue.io accessed on 28 September 2021). Query parameters were selected as Gene expression (L1000 v1.0, Broad Institute, Cambridge, MA, USA), Touchstone, and Individual query. Touchstone serves as a benchmark for assessing connectivity between perturbations. Signatures with a connectivity score >90 (similarity), under compound perturbagens on MCF7 cells, were used further to identify the mechanism of action. Since Memantine and Donepezil are included in Touchstone, we also checked their scores to compare them with Tanshinone IIA and Danshensu.

### 3.6. Experimental Validation

#### 3.6.1. Materials and Chemicals

Danshenxinkun D (M308, 98873-76-8) (purity was higher than 98%) used in the present study was isolated from the roots of *Salvia miltiorrhiza* Bunge and supplied by the QCHENG Bio-Technology (Shanghai, China). Dulbecco’s modified Eagle medium (DMEM) was purchased from Servicebio Co. Ltd. (Wuhan, China); Fetal bovine serum (FBS) was purchased from TransGen Biotech (Beijing, China); Phosphate-buffered saline (PBS), 0.25% trypsin-EDTA (1×), antibiotics (penicillin and streptomycin), dimethyl sulfoxide (DMSO), cell counting kit-8 (CCK-8), and BCA protein assay reagent was obtained from Boster Biol. Tech. (Wuhan, China). H_2_O_2_ was purchased from Sigma Aldrich. Co. Ltd. (Shanghai, China). Acridine orange (AO)—ethidium bromide (EB) staining kit was obtained from Beyotime (Haimen, China); The 5,5′,6,6′-tetrachloro-1,1′,3,3′-tetraethylimidacarbocyanine iodide (JC-1) kit was obtained from Bioscience Technological Co. Ltd. (Wuhan, China); 4′, 6-diamidino-2-phenylindole (DAPI) and Dihydroethidium (DHE) probe were obtained from US Everbright^®^ Inc. (Suzhou, China); The assay kits for malondialdehyde (MDA), superoxide dismutase (SOD), catalase (CAT), and glutathione peroxidase (GSH-Px) were purchased from the Michy Biology (Suzhou, China); Acetylcholine (ACh) was purchased from the Nanjing Jiancheng Bioengineering Institute (Nanjing, China). Salvia miltiorrhiza extract (SME) was prepared by our laboratory based on the herbs from three producing areas (Sichuan, Shandong, and Henan). All other reagents used in the study were of analytical grade.

#### 3.6.2. Animals

A total of 10 healthy male SD rats (180–220 g) were purchased from Si-bei-fu Experimental Animal Co., Ltd. (Beijing, China). Before the experiment, the animals were fed adaptively for one week in a 12 h light/12 h dark cycle environment (20 ± 2 °C) and could take food and water freely. All animals were strictly treated following the International Laboratory Animal Care and Use Code, and were approved by the Animals Care and Use Committee of Chengdu University of TCM (Chengdu, China).

The rats were randomly divided into the SME group (*n* = 8) and the vehicle control group (*n* = 2). SME groups were treated with SME at 8.15 mL/kg, 8.15 mL/kg, and 1 mL/100 g (maximum gavage dose), i.g., three times at 6-h intervals. The vehicle control group was gavaged the same dose of distilled water. One hour after the drug administration, rats in all groups were treated with pentobarbital sodium (150 mg/kg, i.p.). Then blood samples were taken from the abdominal aorta, and the plasma of the same groups was combined, reversed, and mixed evenly. Following a 30-min stand at room temperature, the supernatant was collected and centrifuged at 4 °C 3000 rpm for 10 min. The samples were inactivated in a water bath at 56 °C for 30 min and filtered by a 0.22 μm filter membrane. That is, SME-containing plasma (SMP) and blank plasma were obtained.

#### 3.6.3. Cell Culture and Treatment

The PC12 cells were purchased from Wuhan Pu-nuo-sai Life Technology Co. Ltd. (Wuhan, China) and used throughout the study. PC12 cells were cultured in DMEM supplemented with 10% FBS (*v/v*), penicillin (100 units/mL), and streptomycin (100 μg/mL) at 37 °C in a humidified atmosphere of 5% CO_2_. The PC12 cells were subcultured twice a week, and those in the exponential phase were used in experiments. PC12 cells were pretreated with different concentrations of SMP (4%, 8%, and 16%, diluted with culture medium) for 24 h and then were incubated with 100 μM H_2_O_2_ for another 24 h. The control group was treated with the same amount of DMEM and then stimulated with H_2_O_2_.

#### 3.6.4. Determination of Cell Viability

The CCK-8 assay was employed to detect cell viability to determine the optimal concentration and time of H_2_O_2_ and the optimal intervention concentration of SMP. Briefly, PC12 cells in the logarithmic growth phase were inoculated into 96-well plates (1 × 10^4^ cells/well). Once the cells were fully attached to the wall, H_2_O_2_ at various concentrations for 6–24 h or different concentrations of SMP for 24 h were added for further culture. Subsequently, 10 μL CCK-8 solution was added to each well and cultured for another one hour at 37 °C in the dark. Lastly, the absorbance of each well was detected by using a microplate reader (Bio-Rad, Hercules, CA, USA). Under the 450 nm wavelength, the cell survival rate was calculated. When determining the effects of SMP on H_2_O_2_-induced PC12 cells, we first pretreated the PC12 cells with SMP for 24 h. Then the cells were treated with a determined dose and time of H_2_O_2_, and finally detected the viability of cells after an intervention. The model group was administered with the same amount of DMEM and then stimulated with H_2_O_2_.

#### 3.6.5. Determination of ACh Levels in PC12 Cells

ACh has many functions in the nervous system, such as learning and memory improvement. PC12 cells are a well-established model for the investigation of cholinergic activities. We determined its content with an acetylcholine assay kit to evaluate whether SMP could preserve ACh levels in H_2_O_2_-treated PC12 cells. PC12 cells were incubated with vehicle (as a normal group), H_2_O_2_ (100 µM), or H_2_O_2_ (100 µM) +SMP (4%, 8%, and 16%) as indicated above. After adding reagents to the cells, the supernatant fluid (centrifugation at 3500 r/min for 10 min) was collected to evaluate Ach (550 nm) levels by a Microplate Reader according to the manufacturer’s protocol.

#### 3.6.6. Apoptosis Assay by Dual AO/EB Staining

For the apoptosis of H_2_O_2_-induced PC12 cells, AO/EB double staining could be used to detect the effect of SMP treatment on the morphology. In brief, the cells (1 × 10^5^/well) were inoculated in a confocal laser dish. After the cells were completely attached to the wall, the cells were treated with different concentrations of SMP and H_2_O_2_, as described above. Subsequently, 20 μL AO/EB staining solution was added for 5 min in the dark, and PBS washed the excess staining solution to remove. Finally, a laser confocal microscope (Leica, SP8 SR, Wetzlar, Germany) was used to detect cell apoptosis.

#### 3.6.7. Assessment of Mitochondrial Membrane Potential

The decrease of intracellular mitochondrial membrane potential (MMP, *ΔΨ*m) can be an essential indicator of mitochondrial dysfunction. JC-1 is commonly considered to be an ideal probe for assessing *ΔΨ*m. In the study, PC12 cells were seeded in 6-well plates and treated as described above. Then the cells were incubated with JC-1 at 37 °C in the dark for 15 min. After washing the cells twice with PBS, the fluorescence of cells was measured using laser confocal microscopy (Leica, SP8 SR, Wetzlar, Germany).

#### 3.6.8. Determination of Intracellular ROS Accumulation in PC12 Cells

The DHE fluorescent probe monitored intracellular ROS levels. Briefly, PC12 cells were inoculated in a confocal laser dish (1 × 10^5^/well), treated with different SMP and H_2_O_2_, and incubated at 37 °C for 30 min with a proper amount of the DHE (5 μM) probe. Subsequently, nuclei were counterstained with DAPI, and incubated at room temperature for 5 min. Finally, the staining of the cells was visualized under a confocal laser microscope (Leica, SP8 SR, Wetzlar, Germany).

#### 3.6.9. Determination of MDA, SOD, GSH-Px, and CAT in H_2_O_2_-Induced PC12 Cells

The cells were incubated in 6-well plates for 24 h and given different concentrations of SMP and H_2_O_2_. The supernatants were discarded, then cells were washed with PBS three times. Subsequently, the cells were lysed by RIPA lysis buffer, collected, and centrifuged to obtain the total cell protein. The total protein concentration of the cells was determined using the BCA assay kit. Finally, the levels of MDA and activities of SOD, GSH-Px, and CAT were determined using commercial assay kits according to the manufacturer’s instructions.

#### 3.6.10. UPLC-Q-TOF/MS Analysis

Danshenxinkun D (M308, anti-AD drug candidate in this study) in the SMP was determined by UPLC–Q-TOF/MS analysis. The Acquity UPLC separation system (Waters, Milford, CT, USA) with ZORBAX RRHD Eclipse C18 Column (2.1 × 50 mm, 1.8 µm, Agilent, Palo Alto, CA, USA) was equipped with SYNAPT XS High-resolution mass spectrometry (Waters, Milford, CT, USA). The separation was performed at 35 °C using gradient elution, and water (A) and acetonitrile (B) were used as the mobile phase. The gradient program was set as 0–1 min, 95% A; 1–4 min, 95–80% A; 4–6 min, 80–72%A; 6–8 min, 72–60%A; 8–20 min, 60–42%A; 20–23 min, 42–10%A; 23–23.5 min, 10%A; 23.5–24 min, 10–95%A and 24–27 min, 95%A. The flow rate was 0.4 mL/min, and the injection volume was 2 µL. For mass analysis, nitrogen was applied as auxiliary gas. Capillary voltage: 2.0 kV, Cone voltage: 40.0 V, and Trap Collision Energy: 4.0 V. The mass determination was carried out based on positive and negative scanning modes with the m/z ranging from 50 to 1200.

#### 3.6.11. RT-qPCR Assay

The expression of PSEN1, DRD2, and APP mRNA was examined by RT-qPCR. PC12 cells were pretreated with M308 (100 μg/mL) or SMP (16%), and then stimulated with H_2_O_2_ (100 μM) for 24 h. Total RNA of the PC12 cells was extracted according to the manufacturer’s instruction, and their purity and concentration were determined under their absorbance at 260 and 280 nm. Then 1 μg RNA was reversely transcribed into cDNA using the Master Premix for First Strand cDNA Synthesis Kit (Forgene, Chengdu, China). After adding the SYBR Green PCR Master Mix (Forgene, Chengdu, China), RT-qPCR was performed using qTOWER 3G Q-PCR (Jena, Germany). The reaction process for the RT-qPCR was as follows: 95 °C for 5 s, followed by a cycle of 40 times: 95 °C for 30 s and 60 °C for 30 s. PSEN1, DRD2, and APP gene expressions were normalized to GAPDH and analyzed using the 2^−ΔΔCT^ method. The primers used were as shown in Table 4.

### 3.7. Statistical Analysis

Statistical analyses were performed using one-way ANOVA and multiple comparisons by GraphPad Prism 9 software (GraphPad Software Inc., La Jolla, CA, USA) or t-Test by Excel 2016 (Microsoft, Redmond, WA, USA). *p* < 0.05 was considered the significant level.

## Figures and Tables

**Table 1:** In Silico Molecular docking of the anti-AD compound from Danshen.

Molecule	CAS	Category	Target	Affinity
M001	76822-21-4	Phenolic acids	PLAU	−6.7
M019	20283-92-5	Phenolic acids	APP	−4.4^1^
M029	96574-01-5	Phenolic acids	APP	−5.5
M031	28831-65-4	Phenolic acids	APP	−4.6 ^1^
M081	491-70-3	Phenolic acids	ACHE/APP/MPO	−4.9 ^1^/−4.8 ^1^/−10
M095	21967-41-9	Phenolic acids	ACHE	−6.3
M187	99-50-3	Volatile oil	MPO	−6.3
M206	568-73-0	Tanshinones	NOS3	−12
M208	568-72-9	Tanshinones	DRD1	−10.6
M216	18887-19-9	Tanshinones	ACHE/HTR2C	−5.3/−9.9
M218	146362-71-2	Tanshinones	ACHE	−5.3
M219	142694-58-4	Tanshinones	ACHE/ADAM10/ HTR2A/HTR2C/ NCSTN/PSEN1	−5.2/−6.4/−10.1/ −9.4/−6.1/−8.1
M229	126979-84-8	Tanshinones	ACHE	−5
M230	87205-99-0	Tanshinones	ACHE/HTR2C	−5.3/−10.9
M231	27210-57-7	Tanshinones	ACHE	−4.6 ^1^
M234	105037-82-9	Tanshinones	ACHE/APP/DRD2/ HTR2A/NCSTN/ PSEN1/SLC6A4	−5/−5.1/−10.1/−9.1/−6.1/−7.7/−9.3
M237	119963-50-7	Tanshinones	ACHE/HTR2A/ HTR2C	−4.9 ^1^/−9.5/−9.9
M239	35825-57-1	Tanshinones	ACHE/HTR2A/ HTR2C	−5.2/−9.7/−9.5
M253	189290-30-0	Tanshinones	NCSTN/PSEN1	−5.9/−7.4
M260	76843-23-7	Tanshinones	ACHE/MPO	−5/−9.8
M263	121077-35-8	Tanshinones	ACHE	−4.9 ^1^
M308	98873-76-8	Tanshinones	DRD2/HTR2A/ NCSTN/PSEN1	−9/−8.9/−6.4/−8
M378	13850-16-3	Triterpenoids	SLC6A4	−7.5
M380	4373-41-5	Triterpenoids	SLC6A4	−8.6
M389	4547-24-4	Triterpenoids	SLC6A4	−7.8

^1^ Affinity was stronger than ligand in situ.

**Table 2:** Network scores of anti-AD compound.

Molecule	Count	Score	Z-Value
M234	7	0.6473	5.7989
M308	4	0.5563	4.9337
M081	3	0.0909	0.5071
M219	6	0.0697	0.3055
M237	3	0.0617	0.2294
M239	3	0.0617	0.2294
M216	2	0.0611	0.2244
M230	2	0.0611	0.2244
M260	2	0.0517	0.1351
M095	1	0.0517	0.1347
M218	1	0.0517	0.1347
M229	1	0.0517	0.1347
M231	1	0.0517	0.1347
M263	1	0.0517	0.1347
M019	1	0.0391	0.0151
M029	1	0.0391	0.0151
M031	1	0.0391	0.0151
M253	2	0.0067	−0.2930
M206	1	0.0021	−0.3371
M001	1	0.0002	−0.3546
M208	1	0.0002	−0.3550
M378	1	0.0002	−0.3555
M380	1	0.0002	−0.3555
M389	1	0.0002	−0.3555
M187	1	0.0000	−0.3566
Memantine	2	0.3077	2.5696
Donepezil	4	0.2782	2.2884

**Table 3:** The ACh levels in PC12 cells under different treatments (μg/mL, *n* = 3).

Normal	Model	Low Dose	Middle Dose	High Dose
38.13 ± 4.64 **	12.00 ± 2.03	17.80 ± 2.30	30.97 ± 6.66 **	50.35 ± 3.14 **

Data are mean ± SD. ** *p* < 0.01 vs. model group.

**Table 4:** Primer sequences used for qPCR analysis.

Gene Name	Forward (5′-3′)	Reverse (5′-3′)
PSEN1	TGCACCTTTGTCCTACTTCCA	GCTCAGGGTTGTCAAGTCTCTG
DRD2	GAGCCAACCTGAAGACACCA	GCATCCATTCTCCGCCTGTT
APP	TCCGAGAGGTGTGCTCTGAA	CCACATCCGCCGTAAAAGAATG
GAPDH	AGGTCGGTGTGAACGGATTTG	TCCACCACCCTGTTGCTGTA

## Data Availability

Data are contained within the article and Supplementary Materials.

## Supplementary Materials

The following supporting information can be downloaded at: https://www.mdpi.com/article/10.3390/molecules27144463/s1, Table S1: Plasma absorbed compounds from Danshen; Table S2: Gene sets involved in AD treatment with Danshen; Table S3: CMap analysis result of top 10 compound perturbagens to MCF-7 cell lines; Table S4: Molecular docking parameters (successful docking only).

## Sample Availability

Samples of the compounds are not available from the authors.
